# Blau syndrome polymorphisms in NOD2 identify nucleotide hydrolysis and helical domain 1 as signalling regulators

**DOI:** 10.1016/j.febslet.2014.07.029

**Published:** 2014-09-17

**Authors:** Rhiannon Parkhouse, Joseph P. Boyle, Tom P. Monie

**Affiliations:** a Department of Biochemistry, University of Cambridge, Cambridge, UK; b Department of Veterinary Medicine, University of Cambridge, Cambridge, UK

**Keywords:** BS, Blau syndrome, CAPS, cryopyrin-associated periodic syndromes, EOS, early onset sarcoidosis, HD, helical domain, NACHT, found in NAIP, CIITA, HET-E and TP-1, NF-κB, nuclear factor kappa B, NLR, nucleotide-binding, leucine-rich repeat containing receptor, NOD, nucleotide oligomerisation domain, RIP2, receptor interacting protein 2, SNP, single nucleotide polymorphism, Nucleotide-binding, leucine-rich repeat containing receptor, Nucleotide oligomerisation domain containing 2, Blau syndrome, NACHT, Single nucleotide polymorphisms, Innate immunity

## Abstract

Understanding how single nucleotide polymorphisms (SNPs) lead to disease at a molecular level provides a starting point for improved therapeutic intervention. SNPs in the innate immune receptor nucleotide oligomerisation domain 2 (NOD2) can cause the inflammatory disorders Blau Syndrome (BS) and early onset sarcoidosis (EOS) through receptor hyperactivation. Here, we show that these polymorphisms cluster into two primary locations: the ATP/Mg^2+^-binding site and helical domain 1. Polymorphisms in these two locations may consequently dysregulate ATP hydrolysis and NOD2 autoinhibition, respectively. Complementary mutations in NOD1 did not mirror the NOD2 phenotype, which indicates that NOD1 and NOD2 are activated and regulated by distinct methods.

## Highlights

• NOD2 SNPs that cause Blau syndrome cluster in two regions of the NACHT.
• The ATP/Mg2+ binding pocket cluster are likely to dysregulate ATP hydrolysis.
• SNPs in helical domain 1 are predicted to influence receptor autoinhibition.
• Complementary mutations in NOD1 do not all result in hyperactivation.

## 1 Introduction

Blau syndrome (BS) is a rare autosomal dominant disease manifesting as a triad of symptoms – rashes, uveitis and arthritis – between 3 and 4 years of age. BS is associated with gain-of-function single nucleotide polymorphisms (SNPs) in the NACHT (found in NAIP, CIITA, HET-E and TP-1) domain of the innate immune receptor NOD2 (nucleotide oligomerisation domain containing 2) [1], [2], [3], [4]. Patients with the phenotypically similar disease early-onset sarcoidosis (EOS) possess SNPs in common with BS [4], supporting the suggestion that EOS is a sporadic, rather than familial, version of BS [5]. Loss-of-function SNPs in NOD2 have been strongly associated with the inflammatory bowel condition Crohn’s Disease [6].

NOD2 is a member of the cytosolic NLR (nucleotide-binding, leucine-rich repeat containing) family of pattern recognition receptors [7]. NOD2 is activated by the peptidoglycan component muramyl dipeptide, following which NOD2 engages the adaptor receptor interacting protein 2 (RIP2) to initiate pro-inflammatory signalling pathways involving nuclear factor kappa B (NF-κB) and stress kinases [7], [8], [9]. NOD2 plays an important role in the response to bacterial infection, including the activation of autophagy.

In this work we have analysed 16 currently reported BS or EOS-associated NOD2 SNPs using NF-κB reporter assays to confirm that all but two of these result in a hyperactive form of NOD2. Consistent with an increase in the level of basal signalling the polymorphisms show an increased propensity for NOD2 to relocate to the plasma membrane. Mapping these polymorphisms to a homology model of the NOD2 NACHT shows that they cluster around the helical domain 1 (HD1) and the nucleotide binding pocket. We propose that this results in dysregulation of NOD2 signalling by impacting receptor autoinhibition and nucleotide hydrolysis.

## 2 Results

### 2.1 Blau syndrome associated NOD2 SNPs show basal hyperactivation

**Fig. 1:**
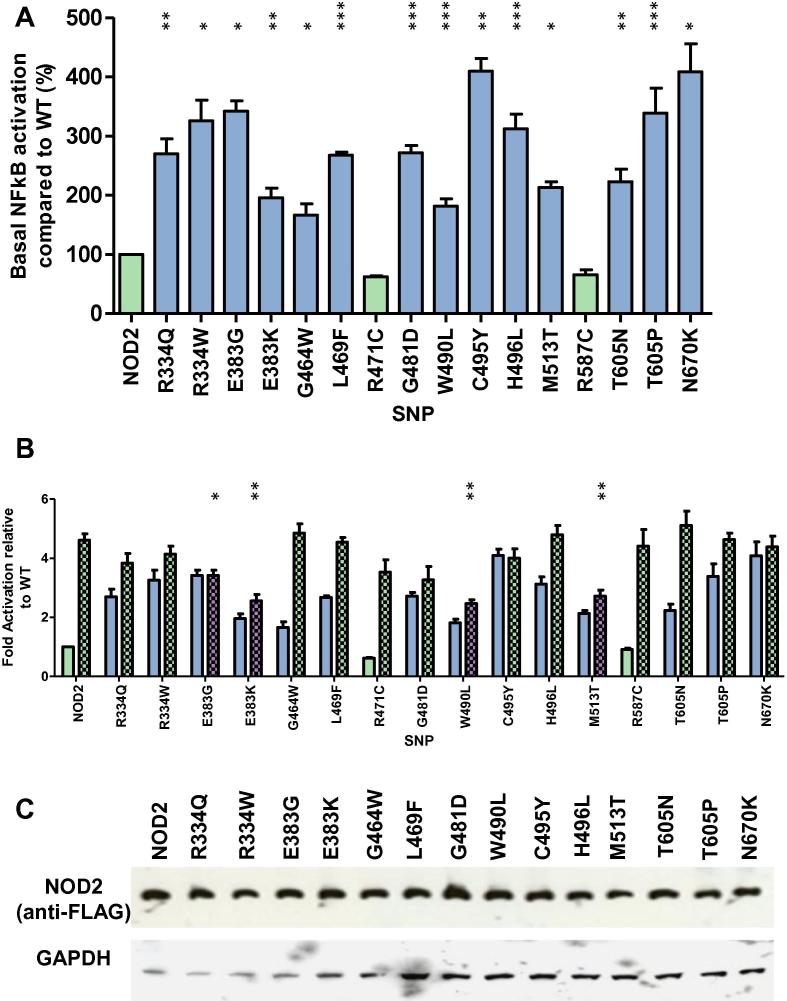
Blau syndrome single nucleotide polymorphisms result in NOD2 hyperactivity. Hyperactive NACHT SNPs were assayed for NF-κB activity in the (A) absence or (B) presence of MDP. Assays were performed in HEK293 cells on 96 well plates. Cells were transfected with 0.1 ng of the relevant pCMV-FLAG-NOD2 plasmid and lysed 24 h later. Graphs are coloured as follows: light green – no significant difference to wildtype; blue – a significant hyperactive response; purple – a significant reduction in the degree of receptor signalling. In panel (B) open bars represent unstimulated samples and chequered bars represent stimulation with 100 ng/ml of MDP. Error bars denote standard error of the mean and statistical significance is denoted by asterisks (^∗^=<0.05, ^∗∗^=<0.01, ^∗∗∗^=<0.001). (C) HEK293 cells in 12 well plates were transfected with 1 μg of the appropriate pCMV-FLAG-NOD2 plasmid. After 24 h, lysates were western blotted with mouse anti-FLAG and mouse anti-GAPDH primary antibodies and goat anti-mouse secondary antibody.

Sixteen NOD2 SNPs associated with BS or EOS (Table 1) were tested for their impact on NOD2 receptor signalling (Fig. 1). All of the eleven SNPS previously reported to result in autoactivation of NOD2-mediated NF-kB signalling were hyperactive in the absence of ligand stimulation (Fig. 1A) confirming the robustness of our reporter assay. Three of the five uncharacterised SNPs (G464W, W490L and T605N) were also hyperactive (Fig. 1A). However, neither R471C nor R587C produced a hyperactive response. None of the SNPs responded in a hyperactive manner following ligand stimulation (Fig. 1B). Whilst most SNPs showed a slightly enhanced signalling response in the presence of MDP, four of them (E383G, E383K, W490L, and M513T), showed a significantly impaired response to ligand compared to the wild-type receptor (Fig. 1B). All of the SNPs expressed at comparable levels to wild-type NOD2 (Fig. 1C).

**Table 1:** NOD2 SNPs investigated in this study reportedly associated with Blau syndrome (BS) or early onset sarcoidosis (EOS). Nd = not determined.

SNP	Disease association	Reported impact on NOD2 signalling	References
R334Q	BS/EOS	Autoactive	[1], [3], [4]
R334W	BS/EOS	Autoactive	[1], [3], [4]
E383G	BS	Autoactive	[27]
E383K	BS/EOS	Autoactive	[23]
G464W	BS	Nd	[29]
L469F	BS/EOS	Autoactive	[1], [3]
R471C	BS/acute myeloid leukemia	Nd	[24], [28]
G481D	BS/EOS	Autoactive	[33]
W490L	BS/EOS	Nd	[23]
C495Y	BS/EOS	Autoactive	[23]
H496L	EOS	Autoactive	[4]
M513T	EOS	Autoactive	[4]
R587C	BS	Nd	[24]
T605N	BS	Nd	[34]
T605P	EOS	Autoactive	[4]
N670K	EOS	Autoactive	[4]

The behaviour of R471C and R587C contrasts with all other BS-associated SNPs and does not fit the hyperactive NOD2 phenotype described for BS raising the possibility that these are not true BS-associated NOD2 SNPs. We compared the sequence of the NOD2 NACHT across fifteen different species, including the relatively divergent platypus and zebrafish (Supplementary Fig. 1). R334, E383, G481, W490, C495 and T605 are conserved across all fifteen species; H496 and M513 differ only in the zebrafish in which they are conservatively changed to tyrosine and valine respectively; whilst G464 and L469 are conserved in the placental mammals but differ in the platypus and zebrafish. In stark contrast R471 and R587 are highly variable across species, consistent with the limited functional impact upon mutation suggesting that they may not actually be disease associated.

### 2.2 NOD1 and NOD2 show different functional phenotypes

It has been proposed that whilst both NOD1 and NOD2 utilise ATP hydrolysis for receptor activation NOD2 additionally requires ATP hydrolysis for receptor deactivation [10], [11]. Consistent with this when the corresponding mutation to E383K (extended Walker-B motif) was made in NOD1 it did not lead to a hyperactive response, instead causing a decrease in NF-κB signalling [10]. We chose to further investigate the parallels between NOD1/2 activation by making mutations in NOD1 corresponding to a broader range of eight different BS/EOS SNPS.

**Fig. 2:**
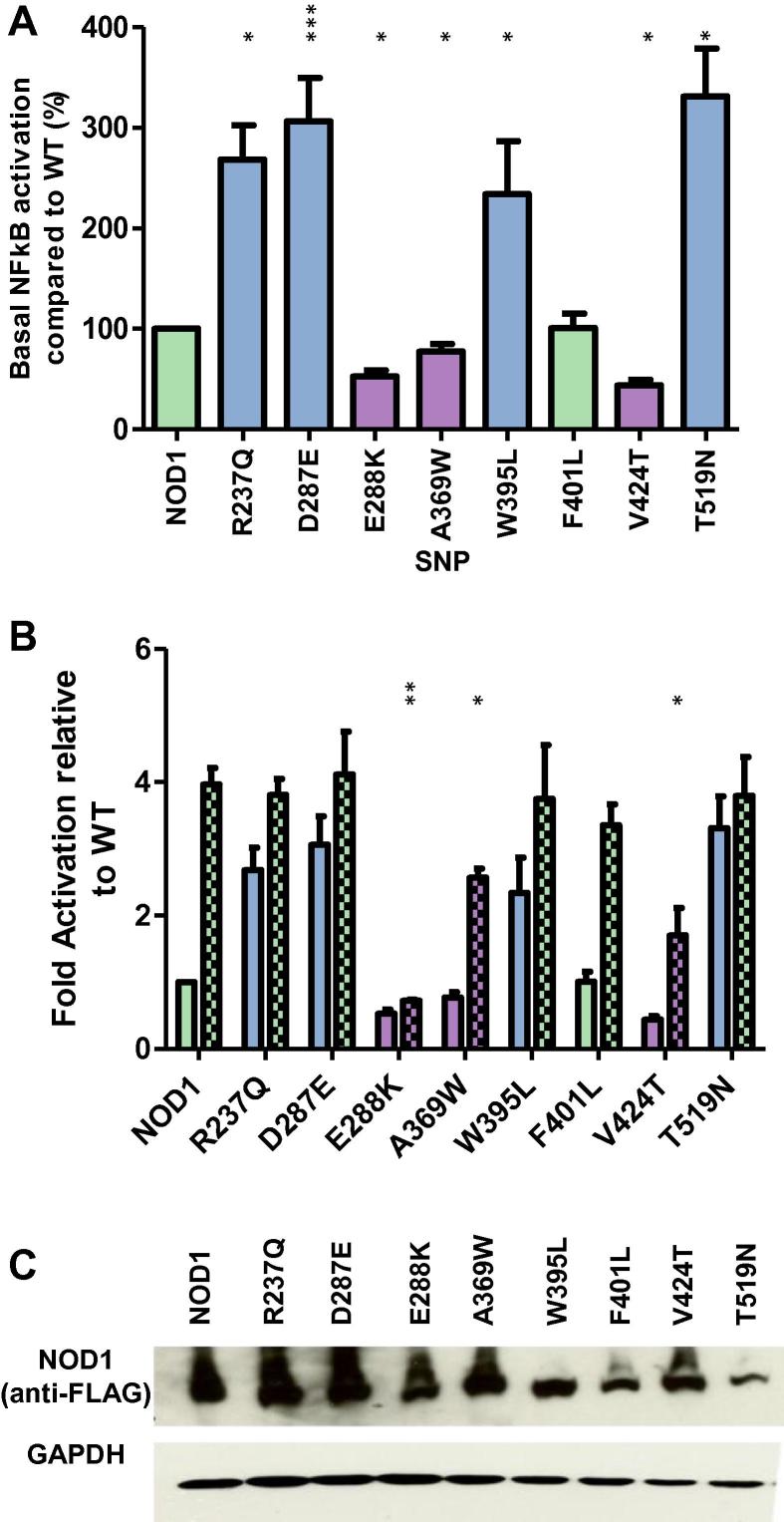
Differential behaviour of complementary mutations to the Blau syndrome polymorphisms in the related NLR protein NOD1. NOD1 NACHT SNPs were assayed for NF-κB activity in the absence (A) or presence (B) of the NOD1 ligand ie-DAP. Assays were performed in HEK293 cells on 96 well plates. Cells were transfected with 0.1 ng of the relevant pCMV-FLAG-NOD1 plasmid (+/− ligand) and lysed 24 h later. Graphs are coloured as follows: light green – no significant difference to wildtype; blue – a significant hyperactive response; purple – a significant reduction in the degree of receptor signalling. In panel (B) open bars represent unstimulated samples and chequered bars represent stimulation with 100 ng/ml of ie-DAP Error bars denote standard error of the mean and statistical significance is denoted by asterisks (^∗^=<0.05, ^∗∗^=<0.01, ^∗∗∗^=<0.001). (C) HEK293 cells in 12 well plates were transfected with 1 μg of the appropriate pCMV-FLAG-NOD1 plasmid. After 24 h, lysates were western blotted with mouse anti-FLAG and mouse anti-GAPDH primary antibodies and goat anti-mouse secondary.

Consistent with the work of Zurek and colleagues NOD1 E288K (equivalent to NOD2 E383K) resulted in a severe decrease in both basal and ligand-stimulated NOD1 NF-κB activity (Fig. 2A and B). Two additional mutations, A369W and V424T (equivalent to NOD2 G464W and M513T) showed a decrease in basal activity but showed a partial response to the addition of ie-DAP (Fig. 2A and B). Meanwhile F401L (NOD2 H496L) behaved in a manner comparable to wild-type NOD1. Interestingly R237Q, D287E, W395L and T519N (corresponding to NOD2 R334Q, D382E, W490L and T605N) all exhibited a hyperactive response (Fig. 2A) and all have wild-type residues identical between NOD1 and NOD2. With the exception of T519N, which had slightly reduced expression, all NOD1 mutants showed a broadly similar expression level to the wild-type receptor (Fig. 2C). These results suggest that NOD1 is less susceptible to SNP induced hyperactivation, consistent with a different mechanism of functional regulation compared to NOD2.

### 2.3 BS SNPs show increased recruitment to the plasma membrane

**Fig. 3:**
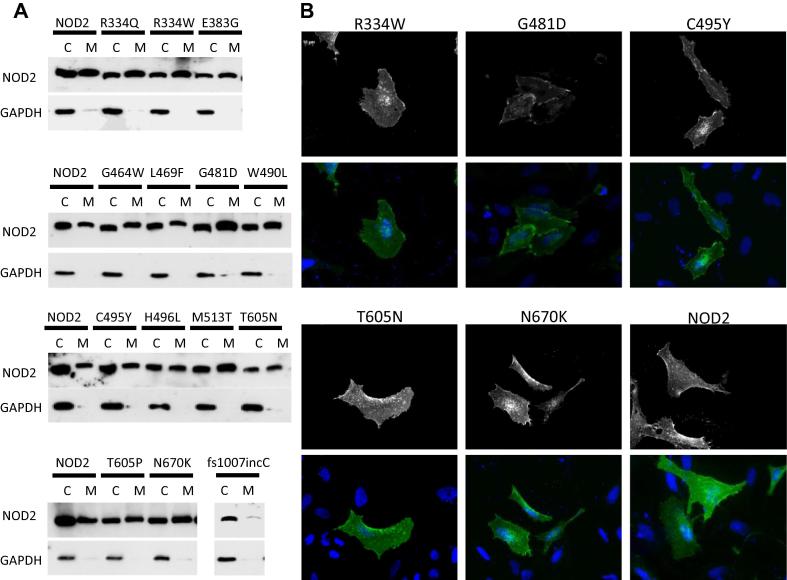
Hyperactive NOD2 Blau syndrome single nucleotide polymorphisms show increased recruitment to the cellular membrane. (A) Subcellular fractionation of HEK293T cells transiently transfected with NOD2 BS/EOS SNPs showed membrane recruitment for all constructs. NOD2 constructs were detected using an anti-FLAG antibody. GAPDH was found only in the cytoplasmic fraction confirming the purity of the separation. (B) Selected NOD2 BS/EOS SNPs were visualised by immunofluorescence following transient transfection of HeLa cells.

Activation of NOD2, and NOD1, has been shown to result in relocalisation of the receptor to the plasma and/or endosomal membranes from where it is believed to form signalling-competent complexes [12], [13], [14], [15], [16]. We used subcellular fractionation to compare the proportion of the NOD2 BS/EOS SNPs in the membrane and the cytoplasmic fractions (Fig. 3A). All hyperactive SNPs were present in the membrane fraction unlike the signalling defective control Crohn’s Disease associated SNP fs1007incC (Fig. 3A). Consistent with previous work [10] the proportion of hyperactive NOD2 at the membrane appeared enhanced in comparison to the wild-type protein. Immunofluorescence on a selection of the hyperactive SNPs confirmed the membrane association of these constructs (Fig. 3B).

### 2.4 Hyperactive SNPS map to distinct regions of the NOD2 NACHT

**Fig. 4:**
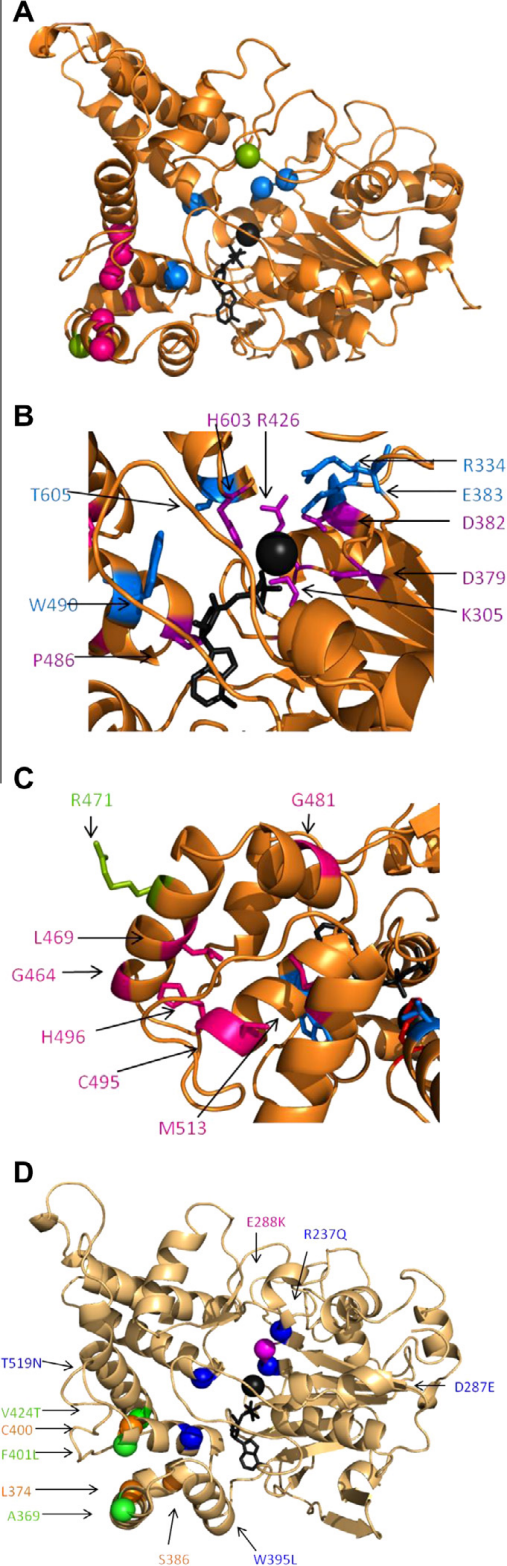
Blau syndrome susceptibility polymorphisms are found in two distinct regions of the NOD2 NACHT. (A) Location of hyperactive/BS SNPs mapped on a model of the NOD2 NACHT. The alpha carbon is represented as a sphere and colour coded as follows: green – signals comparable to wildtype; blue – hyperactive located in ATP/ Mg^2+^ patch; pink – hyperactive located in HD1 patch. (B) Close up of the ATP/Mg^2+^ patch. Conserved residues important in nucleotide binding or hydrolysis are coloured purple. SNPs are coloured blue. R334 and E383 are in close contact with the Mg^2+^ and linked to priming H_2_O for hydrolysis of the ATP molecule. W490 and T605 are not directly in contact with the ATP or Mg^2+^ but T605 is in close proximity to the conserved H603. (C) Close up of the HD1 patch. SNPs located within HD1 are highlighted in pink. These include: G464, L469, G481, C495, H496 and M513. Of these residues L469, C495, H495 and M513 are all internal residues within the domain. G464 and G481 are surface glycines. The wild-type like SNP, R471, is highlighted in green. (D) Location in the NOD1 NACHT of the complementary mutations in NOD1. The alpha carbon is represented as a sphere and colour coded as follows: blue – hyperactive mutations located in the ATP/Mg^2+^ patch; pink – E288 K, an inactive mutant which also resides in the ATP/Mg^2+^ patch; green – mutations in the HD1 patch which gave a wild-type response; orange – mutations which were not made. In each panel the ATP molecule and Mg^2+^ ion are shown in black stick representation and as a black sphere respectively.

It has been suggested that the hyperactivity of the NOD2 E383K BS SNP results from an inhibition of ATP hydrolysis [10]. However, currently there is no proposed mechanism to explain the hyperactivity of all the other BS/EOS SNPs tested. We generated a molecular model of the NOD2 NACHT and mapped the position of the SNPs (Fig. 4A) except for N670 K as the template alignment of the NOD2 HD2 region was too ambiguous to model. The BS/EOS SNPs cluster into two distinct patches: one surrounding the magnesium and ATP binding sites (Fig. 4A and B), and the other on HD1 (Fig. 4A and C). This second patch consists of internal resides within HD1 and two surface glycines (Fig. 4C). The two non-hyperactive SNPs are located on the surface of HD1 (R471C) and in a loop region distal to either patch (R587C). Mapping the corresponding NOD1 mutations onto a model of the NOD1 NACHT domain revealed the same pattern of clustering (Fig. 4D).

**Fig. 5:**
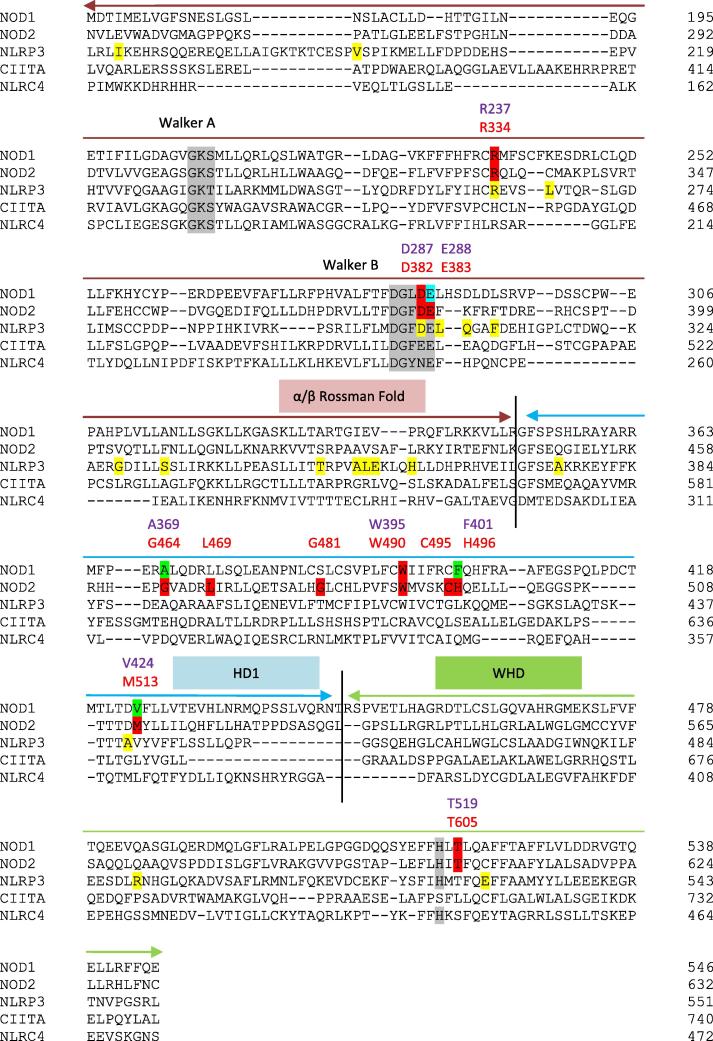
Phyre2 based alignment of NACHT domains from human NOD1, NOD2, NLRP3, CIITA and NLRC4. SNPs in NOD2 associated with BS and leading to a hyperactive response are highlighted and labelled in red. The corresponding mutations in NOD1 are named in purple and highlighted in red (hyperactive), green (like wild-type), or blue (inactive). SNPs in NLRP3 associated with cryopyrin-associated periodic syndromes are highlighted in yellow. The three NACHT sub-domains are marked and conserved functional features are highlighted in grey.

Building on earlier studies [17], [18] alignments of the NOD1 and NOD2 NACHTs with NLRP3 and CIITA, both of which possess hyperactive SNPs; and with NLRC4, the only NLR for which the structure of the NACHT has been solved [19] were made. Reflective of their likely functional role residues, including R334, D382 and E383, around the Mg^2+^/ATP binding patch are predominantly conserved across these NLRs (Fig. 5). This includes two of the NLRP3 SNPs, R260W and D303N (NOD2 R334 and D382), which are associated with cryopyrin-associated periodic syndromes (CAPS) gain of function diseases Muckle–Wells syndrome and Neonatal Onset Multisystem Inflammatory Disease [20]. Other NLRP3 SNPS are mainly spread throughout the α/β Rossman fold sub-domain. The NOD2 SNPs in HD1 show limited conservation with only L469, C495 and M513 being conservatively substituted with hydrophobic residues in the other NLRs. This suggests that the importance of HD1 for the regulation of NOD2 signalling may be unique amongst NLRs.

## 3 Discussion

We have studied SNPs in the NACHT domain of the pattern recognition receptor NOD2 associated with BS and EOS to determine the molecular basis of disease. Hyperactive polymorphisms cluster in two distinct patches in the NOD2 NACHT, one surrounding the ATP/Mg^2+^ binding site and one in the HD1 domain. Complementary mutations in NOD1 did not all result in hyperactivation providing further evidence for a differential mechanism of receptor regulation between NOD1 and NOD2.

The positional grouping of the NOD2 BS/EOS SNPs (Fig. 4) provides a clear functional insight into how they cause disease. SNPs in the Mg^2+^/ATP patch disrupt ATP hydrolysis, a process essential to convert the receptor back to an inactive ADP-bound state [11]. Disruption of ATP binding can deactivate the receptor whereas deregulation of ATP hydrolysis can induce hyperactivation by trapping the receptor in an active state. We propose that SNPs in the ATP/Mg^2+^ patch, including R334Q/W, E383G/K, W490L and T605N/P, inhibit the ATP/ADP cycling required to deactivate NOD2. R334 and E383 are modelled in close contact with the Mg^2+^ ion and are therefore directly important for hydrolysis. Whilst T605 doesn’t directly contact the ATP or Mg^2+^ ion it is close to the conserved histidine, H603, which acts as Sensor II for ATP binding/hydrolysis [17] and which when mutated causes NOD2 hyperactivation [10]. We suggest that the T605N/P SNPs interfere with hydrolysis by spatially disrupting H603.

Meanwhile, SNPs in HD1, and potentially HD2, may interfere with the receptor autoinhibition. HD1 houses the GxP motif which functions to hold the nucleotide in position and HD2 in NLRC4 contacts the LRR in the inhibited conformation [21], [19]. We hypothesise that the HD1 SNPs trap the receptor in an active state by inhibiting formation of the closed, inactive conformation with the LRR folded back onto the NACHT. It is therefore tempting to speculate that the HD1 and HD2 sub-domains act as hinges, changing the conformation of the protein between active and inactive states. Interestingly a region of the murine NAIP proteins encompassing both HD1 and HD2 was shown to be crucial for ligand specificity and activation of the NAIP/NLRC4 inflammasome [22].

Differences between the modes of activation for NOD1 and NOD2 have been previously reported [10]. Whilst Zurek and colleagues did not report any hyperactive NOD1 mutations we have shown that certain mutations in the NOD1 ATP/Mg^2+^ binding patch induce hyperactivity (Fig. 2) supporting an important role for hydrolysis in NOD1 regulation. Clear differences exist in the regulation of NOD1 and NOD2. For example, mutation of the acidic residue, E288, in the extended Walker-B motif of NOD1 results in an inactive response, whereas in NOD2 mutating the equivalent residue, E383, results in a hyperactive receptor. Similarly, mutations in HD1 of NOD1 do not interfere with receptor signalling, whereas the equivalent NOD2 SNPS are hyperactive.

Two SNPs associated with BS, R471C and M587C [23], [24], elicited a wild-type response (Fig. 1). Neither of these SNPs is located in the HD1 or ATP/Mg^2+^ patches identified and lack conservation across different species. Together these cast doubt on the validity of their association with BS. However, NOD2 is the only gene associated with BS, so if these SNPs fail to elicit a hyperactive response maybe other NOD2 SNPs are present in the same patients, although neither study reported this [23], [24]. Comparison of NLRP3 mutations associated with CAPS conditions demonstrated that some of these hyperactive mutations could be located in and around the ATP/Mg^2+^ binding site suggesting that their dysfunction may also relate to altered nucleotide hydrolysis. However, other SNPs were more spread across the α–β Rossman fold suggesting that there may be additional or alternative mechanisms at play in those examples.

The spatial groupings of the NOD2 BS/EOS SNPs we have described, coupled with the functional insight, provide a clear improvement in our understanding of how NOD2 dictates disease pathogenesis in both BS and EOS. Patients with either R334Q or R334W polymorphisms experience the most severe disease symptoms [25] suggesting that disruption of the nucleotide hydrolysis function is more consequential than interference with autoinhibition.

## 4 Materials and methods

### 4.1 Chemicals, antibodies and general methods

Chemical reagents were obtained from Sigma–Aldrich, UK, unless otherwise specified. HEK 293T cells were maintained in DMEM supplemented with 10% Foetal Calf Serum, 100 μg/ml Penicillin/Streptomycin and 2 mM l-glutamine at 37 °C and 5% CO_2_. All transfections were performed using jetPEI™ (Polyplus-Transfection) as per the manufacturers’ instructions. Antibodies used in this work were: rabbit anti-FLAG (F7425, Sigma–Aldrich), mouse anti-FLAG M2 (F3165, Sigma–Aldrich), mouse anti-V5 (ab27671; Abcam), mouse anti-GAPDH (ab9485, Abcam), rabbit anti-Myc (ab9106, Abcam), goat anti-rabbit (ab6721, Abcam) and goat anti-mouse (A4416, Sigma–Aldrich).

### 4.2 Plasmids

pCMV-FLAG-NOD1 and pMV-FLAG-NOD2, encoding N-terminally FLAG-tagged full length NOD1/2 respectively, were a kind gift from Dr. Thomas Kufer [26]. Blau syndrome susceptibility SNPs were identified using published literature [1], [3], [4], [23], [24], [27], [28], [29] and the NCBI SNP database and generated using site directed mutagenesis. Mutant sequences were verified by DNA sequencing of the entire open reading frame. pLuc encoding Firefly luciferase under the control of an NF-κB promoter and phrG encoding Renilla luciferase controlled by a constitutive promoter were kind gifts from Prof Clare Bryant.

### 4.3 NF-kB reporter assays

HEK293T cells in 96 well plates were transfected with: 2 ng pLuc, 1 ng phrG, 0.1 ng wild-type or mutant pCMV-FLAG-NOD2, and made up to 0.1 μg total DNA with empty plasmid. 100 ng/ml MDP or ie-DAP (both Invivogen) was added concomitant with transfection. Cells were lysed 24 h post transfection with 1x passive lysis buffer (Promega) and luminescence measured with a LUMIstar Luminometer (BMG Labtech). Protein expression was checked 24 h after transfection.

### 4.4 Subcellular fractionation

HEK 293 cells seeded in 12 well plates were transfected with 1 μg of wild-type or mutant pCMV-FLAG-NOD2 incubated overnight. Cytosolic and membrane fractions were isolated using a Subcellular Protein Fractionation Kit (Perbio Science UK). Fractions were analysed by SDS–Page and western blotting.

### 4.5 Immunofluorescence

HeLa cells were seeded into 12 well plates containing a sterilised 19 mm diameter glass coverslip, transfected with 1 μg of wild-type or mutant pCMV-FLAG-NOD2 and incubated overnight before washing with 1× PBS and fixing with 4% Paraformaldehyde in PBS for 15 min. Cells were washed again (1× PBS) and permeabilised with 0.4% Triton X-100 (VWR) in 1× PBS for 10 min. Blocking was performed for 20 min using 2.5% goat serum and 1% bovine serum albumin in 1× PBS. Samples were incubated with mouse anti-FLAG primary antibody diluted 1:500 in blocking buffer for 1 h, washed 3 times with 1× PBS and incubated with Alexa Fluor ® 488 goat anti-mouse IgG (Life Technologies) for 1 h. Cells were washed 3 times with 1× PBS, the second wash containing a 1:5000 dilution of 10 mg/ml Hoechst 33258 in PBS to stain the nucleus. Coverslips were mounted onto microscope slides (VWR) using Mowiol mounting solution containing 2.5% 1,4-Diazabicyclo [2.2.2] octane to reduce fading. Cells were visualised using an AXIO Imager.M2 microscope (Carl Zeiss Ltd., Cambridge, UK) and images created using Image J.

### 4.6 Bioinformatics

The NACHT regions of NOD2 from human (*Homo sapien*; NP_071445.1); macaque (*Macaca mulatta*; XP_001084287.1); mouse (*Mus musculus*; NP_665856.2); boar (*Sus scrofa*; NP_001098765.1); cow (*Bos taurus*; NP_001002889.1); dog (*Canis lupus familiaris*; XP_544412.3); guinea pig (*Cavia porcellus*; XP_003477732.1); hamster (*Cricetulus griseus*; XP_003499558.1); horse (*Equus caballus*; XP_001915323.2); rabbit (*Oryctolagus cuniculus*; XP_002721391.1); elephant (*Loxodonta africana*; XP_003416420.1); platypus (*Ornithorhynchus anatinus*; XP_001519938.1); and zebrafish (*Danio rerio*; XP_697924.3) were aligned using Muscle [30]. Phyre2 [31] was used to generate an accurate alignment between the NACHTS of human NOD1 (AAD28350.1), NOD2, NLRP3 (AAI43360.1), CIITA (NP_001273331.1) and NLRC4 (NP_001186068.1).

Generation of a homology model of the NOD2 NACHT covering residues A217-C632 using the NACHT of NLRC4 (PDB: 4KXF; [19]) as a template has been described previously [32]. A homology model of the NOD1 NACHT was generated by the same approach.

## Appendix A Supplementary data

Supplementary data 1 Supplementary Fig. 1: Cross-species comparison of the NOD2 NACHT indicates that Blau syndrome susceptibility single nucleotide polymorphisms are strongly conserved. NOD2 NACHT domains from stated species were aligned using Muscle [30]. Secondary structure is denoted in orange above the sequences and corresponds to NLRC4 NACHT structure (PDB ID: 4KXF; [19]) (β-sheets; arrows, α-helix; ovals). Sequences are highlighted as follows: green - conserved across all species; yellow - some strongly conservative substitutions. Key motifs are highlighted by a black box and labelled. For accession codes see Methods.

Supplementary data 2 This file contains supplementary data consisting of a homology model of the NOD1 NACHT.

Supplementary data 3 This file contains supplementary dataconsisting of a homology model of the NOD2 NACHT.
