# Supplementary material for: Blau syndrome polymorphisms in NOD2 identify nucleotide hydrolysis and helical domain 1 as signalling regulators
Source: FEBS Lett. 2014 Sep 17;588(18):3382–9. doi: 10.1016/j.febslet.2014.07.029 (PMC4158908; doi:10.1016/j.febslet.2014.07.029)
Supplement: Supplementary data 1 — Supplementary Fig. 1: Cross-species comparison of the NOD2 NACHT indicates that Blau syndrome susceptibility single nucleotide polymorphisms are strongly conserved. NOD2 NACHT domains from stated species were aligned using Muscle [30]. Secondary structure is denoted in orange above the sequences and corresponds to NLRC4 NACHT structure (PDB ID: 4KXF; [19]) (β-sheets; arrows, α-helix; ovals). Sequences are highlighted as follows: green - conserved across all species; yellow - some strongly conservative substitutions. Key motifs are highlighted by a black box and labelled. For accession codes see Methods. [file mmc1.docx]

**Supplemental Figure 1**

Mouse FLSTYDGSENLCLEDIYTENILELQTEVGTAGALQKSPAILGLEDLFDTHGHLNRDADTI 268

Hamster FLSTYDGSENLCLEDIYTENILELRTEVGMAGALQKSPAILGLEELFGTHGHLNKDADTV 267

Guinea pig SLSTYDGVENLCLEDIYTENILELRTEVG---SLKKSPSTLGLEELFSTQGHLNEDADTV 314

Human FLSTYDGAETLCLEDIYTENVLEVWADVGMAGPPQKSPATLGLEELFSTPGHLNDDADTV 295

Macaque FLSTYDGAETLCLEDIYTENGLEVWADVGMAGPPQKSPATLGLEELFSTRSHLNDDADTV 299

Rabbit FLSTYDGAENLCLEDIYTENTLEVRTEVGMAGPLHKSPAALGLEELFSPNGHLNEDADTV 311

Boar FLSTYDGAENLCLEEIYTENVLEVRTEGGMTGPPQQSPATLSLGELFSPQGHLNKDADTV 268

Cow FLSTYDGAENLCLEEVYTENVLEIQMEVGMAGPSQQSPTTLGLEELFSTRDHFNKEADTV 268

Dog FLSTYDGAENLCLEEIYTENVLEIRTEMGLARSPQKSPATLSLEELFSTCGHLNEDADTV 347

Horse FLNTYDGAENLCLEEIYTENVLEIRTELGMAGPPQQSPATLGLEELFSTRGHLNKDADTV 268

Elephant FLSTYDGAENLCLEDIYTENVLEVRTNIGMAGPPQQSPATLGLEELFSSQGCLNEDADTV 320

Platypus FLSTYDGMENLCLEDVYTENALEIRKIDGASEPWRKGPAPLGLPDLFDPGGSLNEDADTV 268

Zebrafish FLSTYGGTGRFSLDDIYTDGHLEVMNSSG-------ETTTLGLEDVVGPMGTLNEDADTV 273

*.**.* :.*:::**:. **: * .: *.* ::... . :* :***:

R334Q/W

α/β Rossman Fold

Mouse LVVGEAGSGKSTLLQRLHLLWATGRSFQEFLFIFPFSCRQLQCVAKPLSLRTLLFEHCCW 328

Hamster LVVGEAGSGKSTLLQRLLLLWATGQNFQEFLFVFPFSCRQLQCMAKPLSLKTLLFEHCCW 327

Guinea pig LVVGEAGSGKSTLLQRLHLLWATGRDFQEFLFVFPFSCRQLQCVTKPLSVRTLLFEHCCW 374

Human LVVGEAGSGKSTLLQRLHLLWAAGQDFQEFLFVFPFSCRQLQCMAKPLSVRTLLFEHCCW 355

Macaque LVVGEAGSGKSTLLQRLHLLWAAGRDFQEFLFVFPFSCRQLQCVAKPLSVRTLLFEHCCW 359

Rabbit LVVGEAGSGKSTLLQQVHLLWATGQDFQEFLFVFPFSCRQLQCVARPLSVMTLLFEHCCW 371

Boar LVVGEAGSGKSTLLQQVHLLWASGQAFQEFLFVFPFSCRQLQCLAKSLSLQTLLFEHCCW 328

Cow LVVGEAGSGKSTLLQQLHLLWASGRAFQEFLFVFPFSCRQLQCLVKPLSMRTLLFEHCCW 328

Dog LVVGEAGSGKSTLLQRMHLLWASGRDFQEFLFVFPFSCRQLQCVAKPLSVQMLLFEHCCW 407

Horse LVVGEAGSGKSTLLQQLHLLWAAGRDFQEFLFVFPFSCRQLQCLAKPLSVRALLFEHCCW 328

Elephant LVVGEAGSGKSTLLQQLHFLWAAGRDFQEFLFVFPFSCRQLQCVAKPLSVRTLLFEHCCW 380

Platypus LLVGGAGSGKTTLLQRLVWLWATGRAFQDCLLVFPFSCRQLQRLGKPVSVRTLFFEHCCW 328

Zebrafish LVSGEAGSGKSTLVQRLHLLWAREALLLNTFLLFPFSCRKLNAEHRELSLKELLFLHCCW 333

*: * *****:**:*:: *** : : :::******:*: : :*: *:* ****

Walker A

E383G/K

Mouse P**D**VAQDD**V**FQFLLDHPDRVLLTF**D**GLD**E**FKFRFTD**R**ERHCSPIDPTSVQTLLFNLLQGNL 388

Hamster P**D**VGQHD**V**FQFLLDHPDRVLLTF**D**GLD**E**FKFRFTD**R**ERHCSPTDPTSVQTLLFNLLQGNL 387

Guinea pig P**D**VGQQD**V**FQFLLDHPDCVLLTF**D**GFD**E**FRFKFTDQVRHCSPADPTSVQTLLFNLLQGNL 434

Human P**D**VGQED**I**FQLLLDHPDRVLLTF**D**GFD**E**FKFRFTD**R**ERHCSPTDPTSVQTLLFNLLQGNL 415

Macaque P**D**VDQED**I**FQFLLDHPDRVLLTF**D**GFD**E**FKFRFTDHERHCSPTDPTSVQTLLFNLLQGNL 419

Rabbit P**D**VGQQD**V**FQFLLDHPDRILLTF**D**GFD**E**FKFKFTDHERHCSPTDPTSVQTLLFNLLQGNL 431

Boar P**D**RGQQD**V**FQVLLDHPERILLTF**D**GFD**E**FRFRFTDHERHCCPTAPTSVQSLLFNLLQGNL 388

Cow P**D**LGPQD**V**FQVLLDHPERILLTF**D**GFD**E**FRFRFTDQERHCCPTAPTSVQSLLFNLLQGNL 388

Dog P**D**FGQQE**V**FQFLLDHPNRVLLTF**D**GFD**E**FRFRFSDHERHCSPTDPTSVQNLLFNLLQGNL 467

Horse P**D**LGQQD**V**FQFLLDHPDRVLLTF**D**GFD**E**FRFRFTDGERHCSPTDPTSVQNLLFNLLQGNL 388

Elephant P**D**VGQQD**V**FQFLLDHPDRVLLTF**D**GFD**E**FRFRFTD**R**ERHCSPTDPTSVQNLLFNLLQGNL 440

Platypus P**D**GRQRD**L**FRFILDRPQRVLLTF**D**GLD**E**FRFRFAEGVRHCSPTEPTSVQNLLVNLIQGNL 388

Zebrafish P**D**RNQDE**V**FQFILDHPHLVLFTF**D**GLD**E**FKLGFTDEERHCCPTKQVPIPVLLFNLLQGTL 393

** ::*:.:**:*. :*:****:***:: *:: ***.* ..: **.**:**.*

Extended Walker B

R471C

G464W

L469F

Mouse LKNACKVLTSRPDAV**S**ALLRKFVRT**E**LQLKGFSEEGIQLYLRKHHR**EPG**VADR**L**IQLIQA 448

Hamster LKNAHKVLTSRPDAV**S**ALLRKFVRI**E**CHLKGFSEEGIEQYLRKHHR**EPG**VADR**L**IHLLQA 447

Guinea pig LKNARKVLTSRPDAV**S**ACLRKYIRT**E**CHLRGFSEEGIELYLRKCHH**EPG**VADR**L**IHLLRA 494

Human **LKNARKVVTSRPAAVSAFLRKYIRTEFNLKGFSEQGIELYLRKRHHEPGVADRLIRLLQE** 475

Macaque LKNARKVMTSRPAAV**S**AFLRKYIRT**E**FNLKGFSEQGIELYLRKRHR**EPG**VADR**L**I**R**LLQG 479

Rabbit LKNARKVLTSRPDAV**S**AFLRKYVRT**E**FNLKGFSEEGIELYLRKCHR**EPG**VADR**L**IHLLQT 491

Boar LKNARKVLTSRPDAV**S**ASLRKHVRS**E**LSLKGFSEEGIELYLRKCHR**EPG**VADR**L**ICLLRA 448

Cow LKNARKVLTSRPSAV**S**ASLRKHVRT**E**LSLKGFSEEGIELYLRKRHR**EPG**VADR**L**LCLLRA 448

Dog LKNARKVLTSRPDAV**S**ALLRKYLRL**E**INLKGFSEEGIELYLRKCHR**EPG**VADR**L**I**R**LLKT 527

Horse LKNARKVLTSRPDAV**S**ALLRKYVHT**E**FTLKGFSEEGIELYLRKCHR**EPG**LADR**L**ISLLRA 448

Elephant LKNARKVLTSRPDAVTAFLRKYVCR**E**LHLRGFSEEGIELYMRRHHR**EPG**VADR**L**ICLLKA 500

Platypus MKGSRKVLSSRPEAVTACLRKYVRK**E**VGLRGFSQEAVERFMRKHHG**DP**RVADR**I**V**R**LVGA 448

Zebrafish MKGVMKVVTSRPHAVGPSLKRYLRK**E**VLLKGFSPGGIDCFVKKHYS**DP**AMATR**V**IESVQG 453

:*. **::*** ** . *::.: * *:*** .:: :::: : :* :* *:: :

Sensor 1

C495Y

H496L

W490L

M513T

G481D

HD1

Mouse TSALH**G**LCHLPVFS**W**MVSR**CH**RELLLQ----NRGFPTTSTD**M**YLLILQHFLLHASPPDSS 504

Hamster TSALH**G**LCHLPVFS**W**MVSR**CH**QELLLQ----NGGFPTTSTD**M**YLLILQHFLLHASPPDSY 503

Guinea pig TSVLY**G**LCHLPVFS**W**MVSK**CH**QELLLQ----NGGSPQTSTD**M**YLLILQHFLLHASPPEAV 550

Human TSALH**G**LCHLPVFS**W**MVSK**CH**QELLLQ----EGGSPKTTTD**M**YLLILQHFLLHATPPDSA 531

Macaque TSALH**G**LCHLPVFS**W**MVSK**CH**QELLLQ----EGVSPKTTTD**M**YLLILQHFLLHATSPDSA 535

Rabbit TSALH**G**LCHLPVFS**W**MVSK**CH**QELLLQ----DGGSPKTTTD**M**YLLILQHFLRHASLPDSA 547

Boar TSALH**G**LCHLPVFS**W**MVSK**CH**QELLLQ----GRGSPKTTTD**M**YLLILQHFLLRASPLDSA 504

Cow TSALH**G**LCHLPVFS**W**MVSK**CH**EELLLQ----GRGSPKTTTD**M**YLLILRHFLLHASPLPLA 504

Dog TSALH**G**LCHLPVVS**W**MVSK**CH**QELLLH----GGGSPKTSTD**M**YLLILQHFLLHASPPDSV 583

Horse TSALH**G**LCHLPVFS**W**MVSK**CH**QELLLQ----GGGSPKTTTD**M**YLLILQHFLLHASPPDSA 504

Elephant TSALH**G**LCYLPVFS**W**IVSR**CH**QELLLQ----GGGSLKTTTD**M**YLLILQHFLLHTSPPDTD 556

Platypus TPALR**G**LCHVPVFS**W**IVSR**CH**VELLRLRQGGGGGAPKTMTD**M**YFLIVRHLVLRS--PLEG 506

Zebrafish NTALL**G**LCHIPVFC**W**IVIK**CY**QELLAG----QDGIPQTITD**V**YLLVLQHFFQRKS--SQP 507

...* ***::**..*:* :*: *** * **:*:*:::*:. :

R587C

Winged helix domain

Mouse PLGLGPGLLQSRLSTLLH**L**GHLALRGLAMSCYVFSAQQLQAAQVDSDDISLGFLV**R**AQSS 564

Hamster PLSLGPGLLQSRLSTLLH**L**GHLALQGLALSCYVFSAQQLQEAQVDADDISLGFLV**R**AQNV 563

Guinea pig SRGLVPGLLRSKLPSLLH**L**GRLAFWGLGRCCYVFSAQQLQKAQVDPEDISLGFLV**R**AQGV 610

Human SQGLGPSLLRGRLPTLLH**L**GRLALWGLGMCCYVFSAQQLQAAQVSPDDISLGFLV**R**AKGV 591

Macaque SQGLGPSLLRGRLPTLLH**L**GRLALWGLGMCCYVFSAQQLQAAQVSPDDISLGFLV**R**AKGV 595

Rabbit SQGLGPSLLQGRLPTLLR**L**GQLALWGLGMCCYVFSAQQLQAAQVDPDDISLGFLVQAQGV 607

Boar AQHLGPDLLRGSLPTLLH**L**GHLALWGLGTCCYVFSAEQLQAAHVDSEAVSLGFLV**R**AKSV 564

Cow THGLGPSLIQGRLPTLLH**L**GRLALWGLGTCCYVFSAKQLQAAHVDSEDLSLGFLVLAKRV 564

Dog PHSLGSHLLRGRLPTLLH**L**GWLALWGLGMCCYVFSAKQLQAAHIDDEDISLGFLVHAKTV 643

Horse PGGLEPGLLRGRLPTLLH**L**GQLALWGLGTCCYVFSAKQLQAAHVDSEDISLGFLV**R**AKRA 564

Elephant PRGPGPSLLRGRLPTLLR**L**GHLALWGLGTCCYVFSAKQLQAAQVDTDDIALGFLV**R**AKSV 616

Platypus DRGPAGGVWRGRLPALLR**L**GELALGGLGAGCYVFSAGQLQAAGVSAEDLSLGFLVPSKGS 566

Zebrafish QSGLGKAWLAEHLDTVLK**L**GELALEGLQTSCYVFSGYELQRNRITEQDVGIGFLIYCSDI 567

* ::*:** **: ** *****. :** : : :.:***: ..

T605N/P

Mouse VPGS----KAPLEFLHI**T**FQCFFA**A**FYLAVSADTSVASLKHLFSCGRLGSSLLGRLLPNL 620

Hamster VPGS----KTPLEFLHI**T**FQCFFA**A**FYLAVSADTSAASLRHLFSCGRLGSSLVVRLLPTL 619

Guinea pig VPGS----TAPLEFLHI**T**FQCFFA**A**FYLVLCVDVPKASLRHLFKCRRPGSSPLTRLLPVL 666

Human VPGS----TAPLEFLHI**T**FQCFFA**A**FYLALSADVPPALLRHLFNCGRPGNSPMARLLPTM 647

Macaque MPGS----TAPLEFLHI**T**FQCFFA**A**FYLAFSADVSPALLRHLFNCGRPGNSPMARLLPTL 651

Rabbit VPGS----TAPLEFLHI**T**FQCFLA**A**FYLVLSTDVPTASLRYLFNCRRPGSSPLSRLLPRL 663

Boar VPGG----TPPLEFLHV**T**FQCFFA**A**FYLALSADVPPSSLRHLFHGHRPGSSPLAKVLPKL 620

Cow VPGS----TAPLEFLHI**T**FQCFFA**A**FYLALSADTPPSSLRHLFQDHRPESSPLARVLPKL 620

Dog GPGS----TTPLEFLHI**T**FQCFFA**A**LYLVLSTDVSPSLLRQLFICHGPRSSLLARLLPTT 699

Horse VPGS----APPMEFLHI**T**FQCFFA**A**FYLVLSADVPPSSLRYLFSCGRPGSSLLARLLPAM 620

Elephant VLGS----SAPLEFLHI**T**FQCFFA**A**FYLVLSSDLPPTTLRHLFNCCRTGGSLLARLLPAL 672

Platypus AGGAGGSPEARFEFLHV**T**FQCFLA**A**LYLLLHGDVAPAAVGHLFHRPR-KSRVLELLYPRL 625

Zebrafish SVND----CKRYEFLHI**T**LQCFFA**A**LYVILNRNNDRSAISRLFQPRNRQVSGLSQSCLGQ 623

. ****:*:***:**:*: . : : : ** :

Conserved Histidine

N670K

HD2

Mouse **C**IQGSRVKKGSEAALLQKAE**P**H**N**LQITAAFLAGLLSQQHRDLLAACQVSERVLLQ**R**QARA 680

Hamster **C**IQGSRVRKGSKA**A**LLQKAE**P**H**N**LQITAAFLAGLLSKEHWDLLAACQISKKVLLQ**R**QACA 679

Guinea pig **C**VQSSRIKEGSVA**A**LLQKIE**P**H**N**LHLTAAFLAGLLSQEHQDLLAECQASEKALLQ**R**HACA 726

Human **C**IQASEGKDSSVA**A**LLQKAE**P**H**N**LQITAAFLAGLLS**R**EHWGLLAECQTSEKALL**RR**QACA 707

Macaque **C**IQGSEGKDGSVA**A**LLQKAE**P**H**N**LQITAAFLAGLLS**R**EHWDLLAECQASEKALL**RR**QACA 711

Rabbit **C**VQGSEHKESTVA**A**LLQKTE**P**H**N**LQITAAFLAGLLS**R**EHRDLLAACQASERSLL**RR**RACA 723

Boar **C**VRGSGCKKSSVAHLLQEAE**P**H**N**LQITAASLAGLLSQEHRGLLAECQVSEKALLQ**R**QACA 680

Cow FLRGSRCREGSVA**A**LLQGAE**P**H**N**LQITGAFLAGLLSQEHRSLLAECQASETALL**RR**WDCV 680

Dog **C**VPRSERKEGSLA**A**LLQEAE**P**H**N**LQITAAFLAGLLS**R**EHRGLLAECQANEEALL**R**LQGRA 759

Horse **C**VQGSRCKEDSVESLLQEAE**P**H**N**LQITAAFLAGLLS**R**EHRGLLAKCQGSETALRQ**R**QACA 680

Elephant **C**IQRSESKESSVV**A**WLQKAE**P**H**N**LQITAAFLAGLLS**R**EHRGLLAECQASEKALLQ**R**HTCA 732

Platypus **C**IPAARREEGRPGSLPWGAETV**D**VQLTASFLAGLLSGTNFAPLAESHGS-EALL**R**KRASA 684

Zebrafish **C**MDHSVEES-------HEAETA**N**LQITAQFVSGLLSQRHNNLLLECCPAAVRERNVKQVV 676

: : .. *. ::::*. ::**** : * . . .

Mouse **R**SCLAHSLREHFHSIPPAV**P**GETKSMHAMPGFIWLIRSLYEMQEEQL**A**QE**A**V**R**RLDIGHL 740

Hamster **R**SCLAQSLREHFHSIPPAV**P**GEAKSMHAMPGFIWLIRSLYEMQEEQL**A**QE**A**V**R**RLDIGHL 739

Guinea pig **R**WCLARSLRKHFHCIPPAV**P**GEAKSMHAMPGFVSLIRSLYEMQEEQL**A**RE**A**V**R**GLDIGHL 786

Human **R**WCLARSLRKHFHSIPPAA**P**GEAKSVHAMPGFIWLIRSLYEMQEERL**A**RK**A**A**R**GLNVGHL 767

Macaque **R**WCLARSLRKHFHSIPPAA**P**GEAKSMHAMPGFIWLIRSLYEMQEERL**A**RK**A**A**R**GLNVGHL 771

Rabbit **R**WCLARSLHKHFRSIPPAV**P**GEAKSMHAMPGFLWLIRSLYEMQEERL**A**QE**A**V**R**GLNVEHL 783

Boar **R**WCLARSLHKHFRSIPPAV**P**GEVKSMHAMPGFIWLIRSLYEMQEERL**A**RD**A**V**R**RLNVGHL 740

Cow **R**RCLTRSLREHFRSIPPAL**P**GEAKSMHALPGFLWLIRSLYEMQEERL**A**RE**A**VCRLNVGHL 740

Dog QGCLSRSLHQHFRSIPPAV**P**GEAKSMHAMPGFLWLIRSLYEMQEERL**A**RE**A**V**R**GLTVGHL 819

Horse **R**WCLARSLHKHFHSIPPAVAGEAKSMHAMPGFIWLIRSLYEMQEERL**A**QE**A**V**R**GLDVGHL 740

Elephant **R**WCLARSLRKYFHSIPPGV**P**GEAKSMHAMPGFLWLIRSLYEMQEERL**A**RE**A**V**R**GLDVEHL 792

Platypus **R**KCLARGIERHFRSIPPAV**P**GELKSLHALPAFLWLIKSLYEMQDEGL**A**RR**A**V**R**GFEVEHV 744

Zebrafish KS-LSKRMQRHFKSIPRPVEGEKKSMHAMPSFVWLIKCIYELQDNSI**A**QD**A**MAKLDVEHL 735

: *:: :..:*:.** ** **:**:*.*: **:.:**:*:: :*: * : : *:
